# Suppression of NK Cell Activation by JAK3 Inhibition: Implication in the Treatment of Autoimmune Diseases

**DOI:** 10.1155/2023/8924603

**Published:** 2023-12-08

**Authors:** Wai Chung Wu, Carol Shiu, Tak Keung Tong, Shui On Leung, Chin Wai Hui

**Affiliations:** SinoMab BioScience Limited, Units 303 and 305 to 307, No. 15 Science Park West Avenue, Hong Kong Science Park, Pak Shek Kok, New Territories, Hong Kong

## Abstract

Natural killer (NK) cell is an essential cytotoxic lymphocyte in our innate immunity. Activation of NK cells is of paramount importance in defending against pathogens, suppressing autoantibody production and regulating other immune cells. Common gamma chain (*γ*c) cytokines, including IL-2, IL-15, and IL-21, are defined as essential regulators for NK cell homeostasis and development. However, it is inconclusive whether *γ*c cytokine-driven NK cell activation plays a protective or pathogenic role in the development of autoimmunity. In this study, we investigate and correlate the differential effects of *γ*c cytokines in NK cell expansion and activation. IL-2 and IL-15 are mainly responsible for NK cell activation, while IL-21 preferentially stimulates NK cell proliferation. Blockade of Janus tyrosine kinase/signal transducer and activator of transcription (JAK/STAT) signaling pathway by either JAK inhibitors or antibodies targeting *γ*c receptor subunits reverses the *γ*c cytokine-induced NK cell activation, leading to suppression of its autoimmunity-like phenotype *in vitro*. These results underline the mechanisms of how *γ*c cytokines trigger autoimmune phenotype in NK cells as a potential target to autoimmune diseases.

## 1. Introduction

Natural killer (NK) cell refers to a type of immune cells that play an important role in innate responses to viral infections and cancer [1–3]. In the innate immune system, NK cell does not require prior sensitization for the recognition and killing of tumor cells of various histologic origins and virally infected cells [4]. Two main subpopulations of NK cells are classified by the surface expression of cluster of differentiation (CD)56 and CD16, termed the CD56^bright^CD16− and CD56^dim^CD16+ NK cells, which differ in terms of phenotype, function, and tissue localization [5, 6]. CD56^bright^CD16− NK cells are primarily found in lymphoid tissues with lower cytotoxic activity, but are efficient producers of cytokines such as interferon-*γ* (IFN-*γ*) and cytolytic proteins such as perforin upon stimulation by proinflammatory cytokines like IL-2 and IL-12 [7–9]. In contrast, CD56^dim^CD16+ NK cells are predominantly present in peripheral blood with high cytolytic activity, but are less responsive to cytokine stimulation than the CD56^bright^CD16− population [5].

The development, homeostasis, and regulation of functional activities of NK cells are predominantly modulated by a cytokine family, namely the common gamma chain (*γ*c or CD132) cytokines [10, 11]. The *γ*c chain is a 40-kDa type I transmembrane glycoprotein that acts as a common receptor subunit for the cytokine family that comprises of interleukin (IL)-2, IL-4, IL-7, IL-9, IL-15, and IL-21 [12]. Upon heterodimerization with a proprietary cytokine receptor (IL-2R*β*, IL-4R*α*, IL-7R*α*, IL9R*α*, or IL-21R), which possesses Janus tyrosine kinase (JAK)-1 protein, the JAK3 protein associated with *γ*c receptor is activated to transduce downstream signals through JAK/signal transducer and activator of transcription (STAT) signaling pathway for triggering immune responses against pathogens [13, 14].

Among six *γ*c cytokines, IL-2, IL-15, and IL-21 play indispensable roles in NK cell development and immune functioning. IL-2 and IL-15 share similar functional properties that they both utilize the same receptor components IL-2R*β* and *γ*c to transduce signaling pathways [15]. Both cytokines play important roles in the activation, proliferation, and cytotoxicity of NK cell. Also, these cytokines can convert NK cells from resting to highly cytolytic phenotype with enhanced secretion of perforin and granzymes [16]; they also promote the generation of IFN-*γ* that further enhances the activities of NK cell and macrophage [17–19]. Furthermore, both cytokines have been shown to induce expression of NKG2D (activating) and CD158a/CD158b (inhibitory) receptors on NK cell lymphocyte subsets originating from regional lymph nodes [20]. A more recently discovered *γ*c cytokine, IL-21, is a remarkable regulator of NK cell function. IL-21 is involved in the upregulation of CD16 expression that potentiates the antibody-dependent cellular cytotoxicity (ADCC) activity and costimulation of perforin, granzymes, and IFN-*γ* secretion [10]. Furthermore, costimulation of peripheral blood mononuclear cells (PBMCs) with IL-2 and IL-21 also triggers proliferation of CD56^bright^ NK cells and significantly upregulates the cytotoxicity of CD56^dim^ NK cells, when compared to IL-2 or IL-21 stimulated groups [21].

Autoimmune disease is distinguished by the loss of self-tolerance and presence of autoreactive immune cells in the body. Indeed, several mechanisms have been proposed to elucidate the detrimental role of NK cells in the progression of diseases, including the ability of recognizing stress-induced ligands on self-tissue cells during infection [22], secretion of proinflammatory cytokines to promote tissue damage [23], and the imbalance between NK activating and inhibitory receptors that promote the generation of autoreactive T cells [24]. For example, increased numbers of NK cell have been observed in clinical samples from patients with Crohn's disease or chronic obstructive pulmonary disease [25, 26]. However, evidences have been suggested for NK cells to play protective roles to limit the severity of immunity through the regulation of T and dendritic cells in tissue repair, inhibition of autoreactive T-cell activity, and induced differentiation of regulatory T cells [27]. Although *γ*c cytokines are correlated with the severity of multiple autoimmune diseases (vitiligo, multiple sclerosis, rheumatoid arthritis, celiac disease, psoriasis, alopecia areata, atopic dermatitis, psoriasis vulgaris, systemic lupus erythematosus, Sjogren's syndrome, and type 1 diabetes) in humans [28–50], their effects on NK cell homeostasis during autoimmune diseases remain unclear.

In the current study, IL-2, IL-15, and IL-21 were found to differentially modulate NK cell expansion and activation phenotype in PBMC culture. Through the blockade of JAK/STAT pathway by JAK inhibitors, NK cell expansion and activation were completely abolished. Interestingly, direct blockade of downstream JAK3 activity with anti-*γ*c antibody could suppress *γ*c cytokine-induced NK cell activation but maintains immunity against tumor cells, suggesting that antibody might offer a safer strategy to treat NK cell-mediated autoimmune diseases. Using the isolated primary NK cell as a model, we further demonstrate that long-term *γ*c cytokine-stimulated NK cells exhibit autoimmune phenotype and cytotoxicity against healthy mesenchymal stem cells (MSCs), and the application of anti-*γ*c antibody is able to rescue MSCs. The current study indicates that application of anti-*γ*c antibody might serve as a better strategy to target NK cell-associated autoimmune diseases.

## 2. Materials and Methods

### 2.1. Cells and Reagents

PBMCs (#10HU-003-CR100M) were purchased from iXCells Biotechnologies (San Diego, California, USA). Myelogenous leukemia cell line K562 (CCL-243™) was obtained from ATCC, and bone marrow MSCs (#A15652) were obtained from Thermo Fisher Scientific (Waltham, MA, USA).

PBMCs were cultured in complete ATCC-modified RPMI 1640 Medium (#A1049101), supplemented with 1x penicillin/streptomycin (#15140122) and 10% fetal bovine serum (FBS) (A3160801). Cells were cultured at 2 × 10^6^ cells/ml. K562 cells were cultured in RPMI 1640 Medium (#11875135), supplemented with antibiotics and 10% FBS, at 2 × 10^5^ cells/ml. Bone marrow MSCs were cultured in MesenPRO RS™ Medium (#12746012, Thermo Fisher Scientific) at 5 × 10^3^ cells/cm^2^. All cell cultures were maintained at 37°C in a humidified incubator with 5% CO_2_ atmosphere. All media and reagents for cell culture were purchased from Thermo Fisher Scientific. The sources of cytokines, inhibitors, and neutralizing antibodies are summarized in Table 1.

### 2.2. WST-8 Proliferative Assay

One hundred thousand PBMC cells were seeded into each 96-well plate before treatment. After 3-day cytokine and drug incubation, 10 *μ*l WST-8 reagent (ab228554, Abcam, Cambridge, UK) was added to each well and optical density of each well was measured at 450 nm between 4 and 8 hr using Varioskan LUX Multimode Microplate Reader (Thermo Fisher Scientific). Each group was tested in duplicate.

### 2.3. Primary NK Cells Isolation and Expansion

NK cells were isolated from PBMC using Human NK Cell Isolation Kit II (#130-092-657, Miltenyi Biotec, Bergisch Gladbach, North Rhine-Westphalia, Germany) according to the manufacturer's protocol. Purified NK cells were resuspended in fresh complete NK MACS medium (#130-114-429, Miltenyi Biotec) with 50 ng/ml premium grade IL-2. After 5-day priming with IL-2, cells were expanded with 50 ng/ml premium grade IL-2 and 25 ng/ml IL-21 up to 3 weeks. Percentage of NK cell population was determined by flow cytometry to confirm the NK cell enrichment and expansion following cytokine treatment.

### 2.4. Flow Cytometry Analyses of PBMCs and Purified NK Cells

Sources of antibodies are summarized in Table 1. Cytokine-stimulated PBMCs or purified NK cells were washed with FACS wash buffer (2% FBS in phosphate-buffered saline (PBS)) and then surface stained with CD3 antibody and CD56 antibody to label the CD3-CD56+ NK cell population. Cells were then incubated with antibodies targeting NK surface markers for 30 min at RT.

For intracellular staining, cells were fixed with 4% paraformaldehyde (#158127, Sigma-Aldrich, St. Louis, Missouri, USA) at 4°C for 10 min after surface staining. After washing and incubating in 0.1% Triton-X in PBS at 37°C for 10 min, cells were labeled with antibodies targeting intracellular markers at 37°C for 30 min. Percentage of positively labeled cells was identified by BD FACS Lyric™ Clinical Cell Analyzer (BD Biosciences, New Jersey, USA) as previously described [51]. Unstained control without antibody incubation was used for gating.

### 2.5. Western Blot (WB)

Antibodies for WB were purchased from CST (Danvers, Massachusetts, USA) or Sino Biological, as summarized in Table 1. Total proteins were extracted from the PBMC in RIPA lysis buffer (#20-188, Millipore, Burlington, Massachusetts, USA) with inhibitor cocktail (#78440, Thermo Fisher Scientific) after 15 min cytokine incubation. Protein concentration was measured by Pierce™ BCA Protein Assay Kit (#23225, Thermo Fisher Scientific) and then diluted in NuPAGE™ LDS Sample Buffer (#NP0007, Thermo Fisher Scientific) supplemented with 2-mercaptoethanol (#1610710, Bio-Rad, Hercules, California, USA). After boiled at 95°C for 10 min, protein lysates (20–40 mg/lane) were separated through electrophoresis and blotted on the nitrocellulose membrane (GE10600001, Sigma-Aldrich). Membrane was blocked with 5% nonfat milk (#1706404, Bio-Rad) and incubated with primary antibodies at 4°C overnight. Next day, membrane was washed with PBST and incubated with horseradish peroxidase (HRP)-conjugated secondary antibodies for 60 min at RT. Protein bands were visualized using ECL substrate kit (#34580, Thermo Fisher Scientific) in the ChemiDoc Imaging System (Bio-Rad).

### 2.6. Cytotoxicity Assays to K562 and MSC

For cell-to-cell cytotoxicity assay, PBMCs or overnight starved purified NK cells were pretreated *γ*c antibody for 1 hr, followed by single cytokine (IL-2/IL-15/IL-21) or cytokine cocktail stimulation for 3 days. K562 and MSCs (target cells) were counted and 1 × 10^6^ cells were labeled with 100 *µ*M of CFSE (#423801, BioLegend) for 10 min at 37°C. Labeled target cells were washed and resuspended in complete ATCC-modified RPMI medium. Prestimulated PBMCs or purified NK cells (effector cells) were introduced to target cells in different E : T ratios. The cell mixture was harvested after 4 hr incubation, washed, and centrifuged. Cells were then resuspended in 50 ng/ml propidium iodide (PI) (#P1304MP, Thermo Fisher Scientific) and analyzed with FACS analysis. CFSE- and PI-labeled target cell populations were quantified to determine cytotoxicity levels of effector cells.

For soluble factor secretion cytotoxicity assay, PBMCs or overnight starved isolated NK cells were pretreated with *γ*c antibody and stimulated with single cytokine or cytokine cocktail as same as the condition of cell-to-cell cytotoxicity assay above. Supernatant was harvested from PBMC and NK cell culture after stimulation for 3 days. K562 was counted and 1 × 10^6^ K562 cells were incubated with the supernatant in 1 : 1 ratio for 16 hr at 37°C. Treated K562 cells were washed, centrifuged, and resuspended in 50 ng/ml PI. The labeled cells were analyzed with FACS analysis. PI-labeled K562 cell population was investigated to determine soluble factor-mediated cytotoxicity.

### 2.7. ELISA

ELISA assay is the golden standard for determining cytokine level in the clinical and laboratory samples [52]. After cytokine stimulation, PBMC and NK cell culture supernatant was harvested and ELISA assays were performed according to manufacturer protocols. DuoSet ELISA measuring human granzyme A (#DY2905-05), granzyme B (#DY2906-05), IFN-*γ* (#DY285B), IL-4 (#DY204-05), IL-5 (#DY205), IL-6 (#DY206), IL-10 (#DY217B05), IL-13 (#DY213), and IL-22 (#DY782-05) were obtained from R&D Systems (Minneapolis, MN, USA). Human perforin-coated ELISA (#BMS2306) was obtained from Thermo Fisher Scientific.

### 2.8. Statistical Analyses

The signal intensities of protein bands in WB were determined by ImageJ (National Institutes of Health, Bethesda, Maryland, USA). Data from flow cytometry were quantified by FlowJo (version 10, BD, Ashland, OR, USA). Statistical analyses were conducted by Prism (version 8, GraphPad Software, Boston, MA, USA). *p*  < 0.05 is considered to be statistically significant between groups.

## 3. Results

### 3.1. JAK/STAT Pathways Were Triggered by IL-2, IL-15, and IL-21 in PBMCs

**Figure 1:**
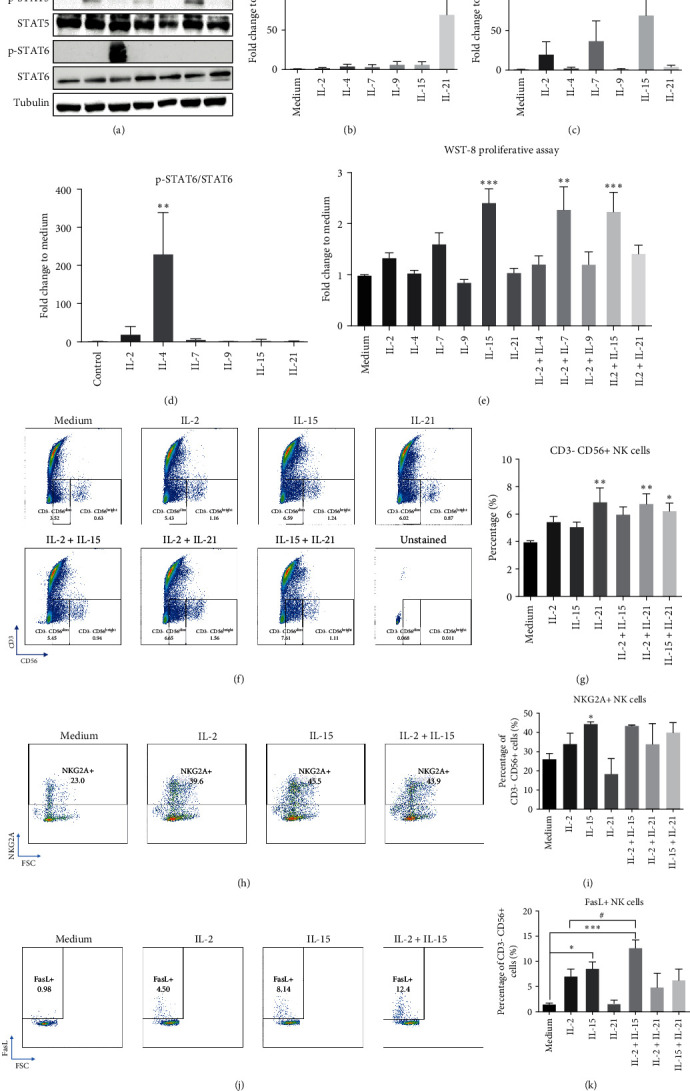
*γ*c cytokines activated JAK/STAT pathways and modulated NK homeostasis in PBMC culture. (a) PBMCs were treated with *γ*c cytokines (50 ng/ml) for 15 min and harvested for western blot analysis. All cytokines, except IL-9, induced phosphorylation of STAT proteins. (b–d) Quantification demonstrated that each cytokine (except IL-9) could stimulate one major STAT phosphorylation in PBMC culture. (e) WST-8 proliferation assay indicated that 3-day treatment of IL-15, IL-2/IL-7, and IL-2/IL-15 (50 ng/ml for each cytokine) significantly induced proliferation of PBMCs. (f) Representative graphs of CD3−CD56+ (CD56^bright^ and CD56^dim^) expressing cells demonstrated 3-day treatment of *γ*c cytokine (IL-2 : 20, IL-15 : 20, and IL-21 : 25 ng/ml) induced NK cells expansion as consistent to WST-8 assay. (g) Quantification demonstrated that IL-21, IL-2/21, and IL-15/21 significantly induced NK cell expansion in PBMC culture. Representative images of 3-day treatment of IL-2 (20 ng/ml), IL-15 (20 ng/ml), and IL-2/15 (20 ng/ml) induced (h) NKG2A+ and (j) FasL+ expression in NK cells. IL-15 and IL-2/IL-15 significantly induced (i) NKG2A and (k) FasL expression, in which IL-2/IL-15 demonstrated synergetic effects in FasL expression. For above experiments, PBMCs were treated with corresponding cytokines with indicated time.  ^*∗*^*p* < 0.05,  ^*∗∗*^*p* < 0.01, and  ^*∗∗∗*^*p* < 0.001 were compared to controls using Dunnett's multiple comparisons test following one-way ANOVA.  ^#^*p* < 0.05 was compared to indicated group by Student's *t*-test. *N* = 3 for (a–e), *N* = 4 for (f–k).

Biological effect of *γ*c cytokines on JAK/STAT pathway in PBMC was investigated through WB analysis. Results showed that each *γ*c cytokine except IL-9 activated one major STAT protein in the culture (Figure 1(a)). STAT phosphorylation was relatively weak in IL-9-treated PBMCs and quantification confirmed the results (Figure 1(b)–1(d)). Proliferation of PBMC was then measured by WST-8 assay after 3-day cytokine treatment. IL-2, IL-7, and IL-15 all promoted PBMC proliferation, and the strongest effect was observed in IL-15 stimulation (Figure 1(e)). Moreover, IL-2 and IL-7 cotreatment could further promote PBMC proliferation in the culture (Figure 1(e)), suggesting the synergistic effect of *γ*c cytokines in PBMC proliferation and STAT5 phosphorylation as a potential indicator for measuring proliferative activity in PBMC culture.

### 3.2. IL-2, IL-15, and IL-21 Differentially Modulated NK Cell Expansion and Phenotype

Next, expansion of immune cell type after 3-day cytokine treatment was investigated through flow cytometry analyses. IL-2 and IL-7 treatment could induce T-cell expansion (data not shown), while IL-21 could increase NK cell population (CD3-CD56+) significantly (Figure 1(f)). Moreover, combined treatment of IL-2/IL-15 showed a trend to synergistically increase NK cell population in PBMC culture (Figure 1(g)). The effects of three *γ*c cytokines on shaping NK cell phenotype were then studied by surface receptor expression analyses. CD94/NKG2A is regarded as an inhibitory receptor for immune checkpoint in NK cells. NKG2A recognizes HLA class I and E on normal cells to inhibit NK cell activity, thus suppressing NK cell-mediated autoimmunity. Although NKG2A+ NK cells function to identify self and nonself-cells in the body, uncontrolled expression of NKG2A is also an indication of NK cell exhaustion [53, 54]. FasL is a type II transmembrane protein that specifically binds to FasL receptors expressing on target cells to induce cytotoxicity [55, 56]. Data indicated that IL-15 could induce NKG2A expression (Figures 1(h) and 1(i)), while IL-15 alone and IL-2/IL-15 combined treatment contributed to FasL upregulation on NK cells (Figures 1(j) and 1(k)), suggesting these two cytokines may play alternative roles in modulating and activating NK cell in a healthy PBMC culture.

### 3.3. IL-2 and IL-15 Contributed to NK Cell Activation in the PBMC Culture

**Figure 2:**
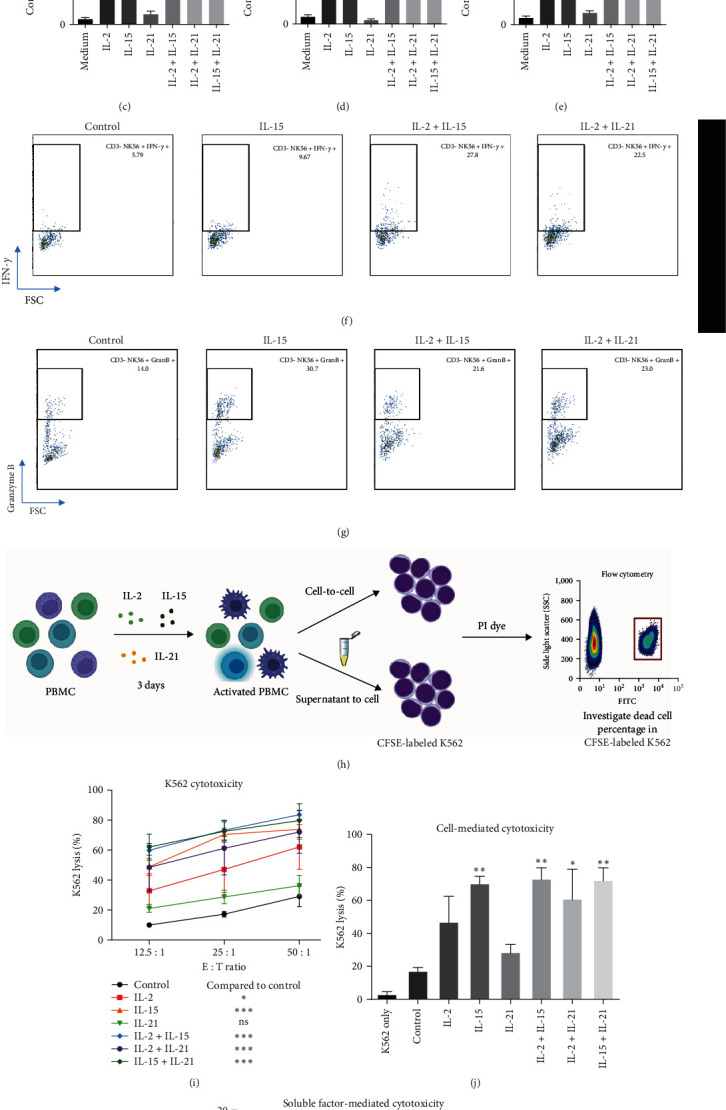
IL-2 and IL-15 contributed to NK cell activation and K562 cytotoxicity. Supernatants were harvested for ELISA, while PBMC cells were harvested for FACS analysis after 3-day cytokine (IL-2 : 20, IL-15 : 20, and IL-21 : 25 ng/ml) incubation. Differential or combinatorial treatment of IL-2- and IL-15-induced production of (a) IFN-*γ*, (b) IL-6, (c) perforin, (d) granzyme A, and (e) granzyme B, respectively. Representative images of intracellular (f) IFN-*γ* and (g) granzyme B in gated NK cells after 3-day cytokine challenges. (h) Schematic diagram for NK cell-mediated cytotoxicity assay with FACS analysis. A 3-day cytokines (IL-2 : 20, IL-15 : 20, and IL-21 : 25 ng/ml) stimulated PBMCs or supernatants were introduced to K562 for studying cell-mediated or soluble factor-mediated cytotoxicity assay, respectively. (i) All studied groups, except IL-21, enhanced K562 lysis significantly compared to control. (j) In E : T ratio of 25 : 1, IL-15, IL-2/IL-15, IL-2/IL-21, and IL-15/IL-21 treated PBMCs significantly lysed K562 lysis cells. (k) Supernatants harvested from IL-15 and IL-2/IL-15 treated PBMCs significantly lysed K562 lysis cells.  ^*∗*^*p* < 0.05,  ^*∗∗*^*p* < 0.01, and  ^*∗∗∗*^*p* < 0.001 were compared to medium or control groups using Dunnett's multiple comparisons test following one-way ANOVA for (a–e) and (j and k).  ^*∗*^*p*  < 0.05 and  ^*∗∗∗*^*p* < 0.001 were compared to control using Tukey's multiple comparisons test following two-way ANOVA for (i). ^##^*p*  < 0.01 was compared to indicated group by Student's *t*-test. *N* = 3 for all groups.

Secretion of perforin, IFN-*γ*, and granzymes are classified as the markers for NK cell activation during infection, tumor development, and autoimmunity progression [7, 57–60]. Thus, we studied whether *γ*c cytokines can modulate the production of these soluble factors to evaluate the NK cell activation status. Similar to previous results, IL-2 and IL-15 significantly induced secretion of IL-6, IFN-*γ*, perforin, and granzymes A/B, in which IL-2/IL-15 cotreatment showed synergistic effect on granzyme B and IFN-*γ* production by PBMC as analyzed by ELISA (Figure 2(a)–2(e)). Although IL-21 alone did not induce secretion of all studied cytotoxic soluble factors, IL-2/IL-21 showed synergistic effect on granzyme B secretion from PBMC culture (Figure 2(e)). To confirm if NK cell is one of the main cell types for soluble factor secretion, intracellular levels of those factors were measured by FACS analysis. Consistent to ELISA results, individual IL-2 and IL-15 treatment could trigger intracellular IFN-*γ* and granzyme B expression in the CD3-CD56+ NK cells (Figures 2(f) and 2(g)), suggesting that NK cell was one of the cell types responsible for granzyme B and IFN-*γ* secretion. To further confirm whether two cytokines could induce activation of multiple NK cell subpopulation, cytokines involved in shaping natural killer type (NK)1/NK2/NK3/NKr cell phenotypes [61, 62] were measured. ELISA results demonstrated that in PBMCs culture, NK2 (IL-13), NK3 (transforming growth factor-*β* (TGF-*β*)), and NKr (IL-10) cytokines were only increased in IL-15/IL-21-treated groups only (*Supplementary 1*, data not shown). By calculating the NK1 (IFN*γ*-producing)/NK2 ratio through dividing IFN-*γ* and IL-6 level by IL-5 level for each individual group, it was identified that the presence of IL-15 or IL-2/IL-21 could predominantly trigger NK1 phenotype in the PBMC culture (*Supplementary 1*).

### 3.4. Prestimulated NK Cells Are Responsible for K562 Lysis

The upregulation of activating receptors and intracellular expression of cytotoxic factors in NK cells potentially led to cytotoxicity on target cells. This phenomenon was studied by determining K562 lysis (PI + cells) either coculture with *γ*c cytokine-activated PBMCs or supernatant harvested from activated PBMC cultures (Figure 2(h)). For cell-mediated cytotoxicity, the presence of IL-15 in all studied PBMC groups triggered significant cytotoxicity against K562 at all studied E : T ratios and the optimal ratio was 25 : 1 (Figures 2(i) and 2(j)). Consistent to cell-mediated cytotoxicity, supernatant harvested from PBMCs with IL-15 or IL-2/IL-15 treatment also led to significant K562 lysis (Figure 2(k)). These results further confirmed that IL-2 and IL-15 play vital roles in promoting NK cell activation and cytotoxicity, while IL-21 preferentially promotes NK cell expansion in the PBMC culture.

### 3.5. JAK/STAT Inhibitor Totally Abolished NK Expansion and Activation

**Figure 3:**
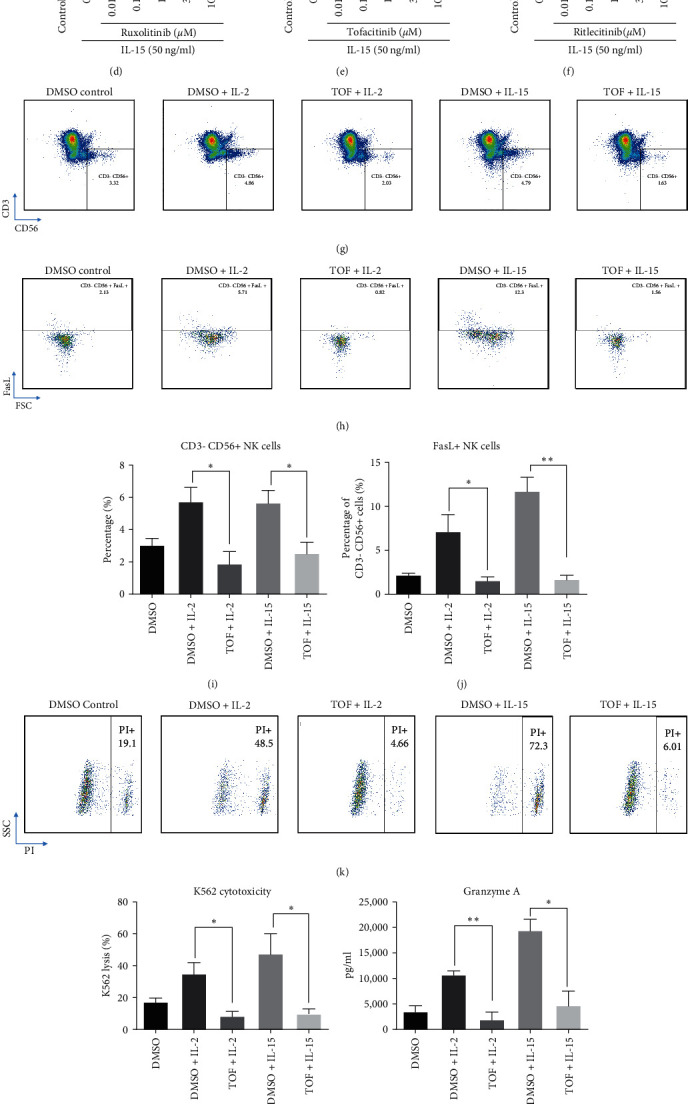
JAK/STAT inhibitors completely attenuated *γ*c cytokines effects on NK cells expansion, activation, and cytotoxicity. PBMCs were pretreated by JAK inhibitors for 1 hr, followed by *γ*c cytokines (50 ng/ml) stimulation for 3 days. Ruxolitinib (JAK1/2), tofacitinib (JAK3), and ritlecitinib (JAK3) inhibited (a–c) IL-2- and (d–f) IL-15-induced proliferation in PBMC culture in dose-dependent manner, respectively. (g and h) Representative images of FACS analysis demonstrating tofacitinib suppressed *γ*c cytokines (50 ng/ml) induced CD3−CD56+ NK cells and FasL+ NK cell population and (i and j) quantification confirmed the results. (k and l) Representative images of FACS analysis showing tofacitinib suppressed IL-2 or IL-15 induced cytotoxicity against K562 and quantification confirmed the results. (m–o) Tofacitinib also eradicated IL-2- and IL-15-induced secretion of granzyme A, Granzyme B and IFN-*γ* secretion in the culture.  ^*∗*^*p*  < 0.05,  ^*∗∗*^*p*  < 0.01, and  ^*∗∗∗*^*p* < 0.001 were compared to control or DMSO control using Dunnett's multiple comparisons test following one-way ANOVA. ^###^*p*  < 0.001 was compared to control by Student's *t*-test. *N* = 3 for all groups.

IL-2 and IL-15 could stimulate several downstream signaling pathways for NK proliferation, activation, and immune functions [63–67]. Among these pathways, JAK/STAT pathway is important for NK cell expansion and activation [68]. This phenomenon was explored using three JAK inhibitors in the PBMC culture according to our previous data [69]. All studied JAK inhibitors could significantly suppress immune cell expansion in the presence of IL-2 and IL-15 dose dependently (Figure 3(a)–3(f)). Low-dose (1 *µ*M) tofacitinib was selected for following experiments to fit with the *γ*c downstream pathway (JAK1/JAK3).

Flow cytometry analyses confirmed that blockade of JAK/STAT pathway by tofacitinib attenuated IL-2- and IL-15-induced expansion of NK cells in the PBMC culture (Figures 3(g) and 3(i)), as well as complete suppression of FasL expression on NK cells (Figures 3(h) and 3(j)). Functionally, tofacitinib totally abolished cell-mediated cytotoxicity against K562 tumor cells (Figures 3(k) and 3(l)) and release of cytotoxic factors (Figure 3(m)–3(o)). Altogether, these data suggested that JAK/STAT pathway is essential for NK cell expansion and activation during IL-2 or IL-15 stimulation and blockade of JAK/STAT pathway potentially suppresses acute NK cell activation.

### 3.6. Blocking *γ*c Receptor Resulted in Attenuation of NK Cell Activation Via Suppressing Cytotoxic Factors Secretion

As tofacitinib preferentially suppressed JAK1 and JAK3 activation in the cells and numerous side effects were observed after JAK1 inhibition in clinical trials [70], we hypothesize if specific blockade of JAK1 and JAK3 activation by neutralizing antibodies against respective receptors could provide comparable efficacy to suppress NK cell activation in our system. Through treating preactivated PBMC cultures with anti-CD25 or anti-CD122 neutralizing antibodies, the data demonstrated that NK cell activation could be attenuated, while NK cell population and cytotoxicity against tumor cells could be retained (*Supplementary 2* and *3*), suggesting that antibody treatment might be a safer strategy for JAK inhibition.

**Figure 4:**
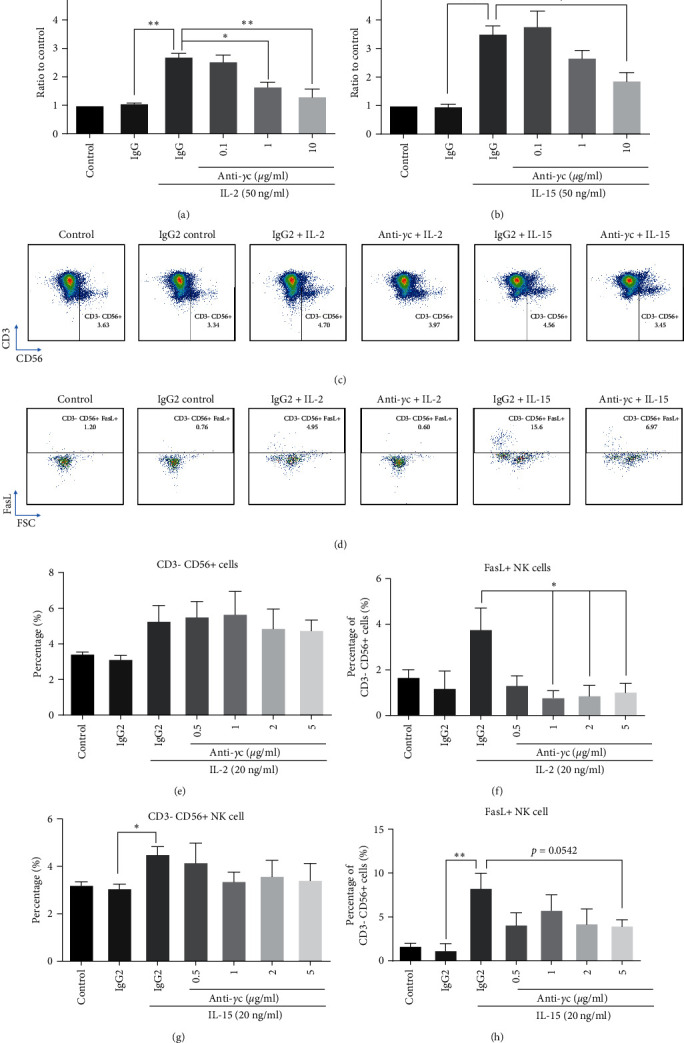
Anti-*γ*c antibody attenuated NK activation only while maintained NK cell survival. PBMCs were pretreated by anti-*γ*c (5 *µ*g/ml) for 1 hr, followed by IL-2 (50 ng/ml) and IL-15 (50 ng/ml) stimulation for 3 days. (a and b) Anti-*γ*c attenuated IL-2- and IL-15-induced proliferation on PBMC culture. Representative images of FACS analysis showing that anti-*γ*c suppressed IL-2- and IL-15-induced (c) CD3−CD56+ NK cells and (d) FasL+ NK cell population. (e and g) Quantification demonstrated that anti-*γ*c did not alter *γ*c cytokines-induced CD3CD56^+^ NK cell population, but significantly suppressed (f) IL-2 and (h) IL-15 induced FasL+ NK cell population.  ^*∗*^*p*  < 0.05,  ^*∗∗*^p  < 0.01, and  ^*∗∗∗*^*p*  < 0.001 were compared to isotype control by Student's *t*-test, *N* = 4.  ^*∗*^*p* < 0.05,  ^*∗∗*^*p*  < 0.01, and  ^*∗∗∗*^*p*  < 0.001 were compared to isotype control with cytokine treatment using Dunnett's multiple comparisons test following one-way ANOVA. *N* = 4 for all groups.

**Figure 5:**
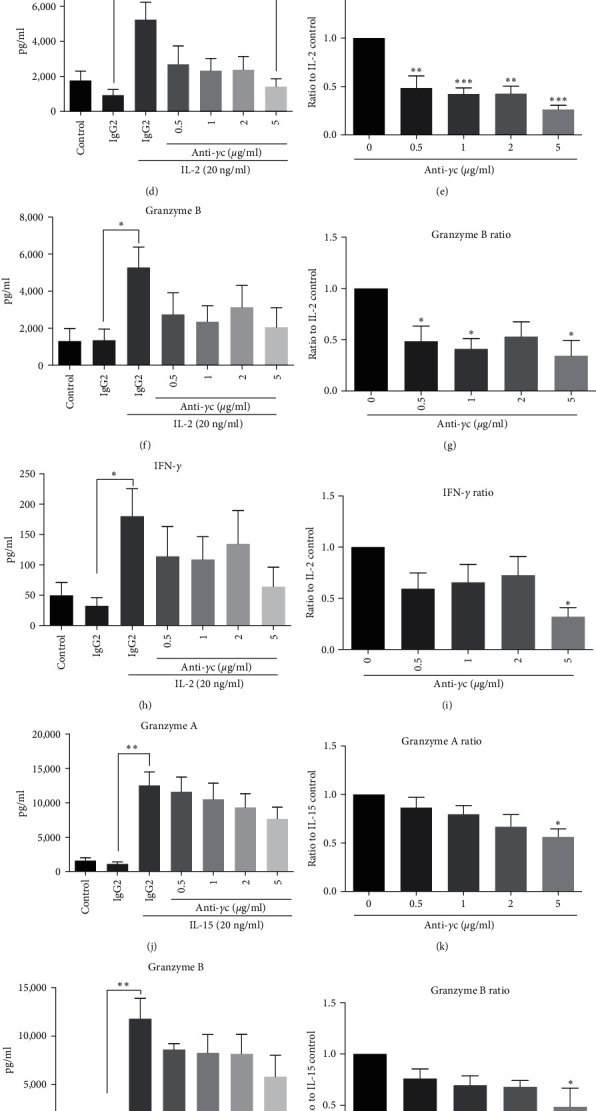
Anti-*γ*c antibody suppressed secretion of cytotoxic factors but maintained NK cells antitumor activities. (a) Representative images of FACS analysis showing anti-*γ*c antibody suppressed cytokines-induced cytotoxicity against K562. Quantification demonstrated anti-*γ*c antibody suppressed (b) IL-2 induced cytotoxicity against K562 significantly, but not for (c) IL-15. (d–m) Anti-*γ*c attenuated IL-2-induced secretion of granzyme a and granzyme B significantly at different doses, while only inhibiting IL-15-induced secretion at 5 *µ*g/ml. (h–o) Anti-*γ*c only attenuated IL-2- and IL-15-induced secretion of IFN-*γ* at 5 *µ*g/ml only. Inhibitory ratio is calculated via dividing values obtained from antibody treated groups over values from isotype controls in the presence of either IL-2 or IL-15.  ^*∗*^*p*  < 0.05,  ^*∗∗*^*p* < 0.01, and  ^*∗∗∗*^*p*  < 0.001 were compared to isotype control by Student's *t*-test, *N* = 4.  ^*∗*^*p*  < 0.05,  ^*∗∗*^*p*  < 0.01, and  ^*∗∗∗*^*p*  < 0.001 were compared to isotype control with cytokine treatment using Dunnett's multiple comparisons test following one-way ANOVA. *N* = 4 for all groups.

Next, the effect of specific JAK3 inhibition by anti-*γ*c antibody was compared to anti-CD25 and anti-CD122 antibodies in restoring normal NK cell phenotype in the presence of cytokine stimulation. In concordance with previous data, anti-*γ*c antibody could significantly reduce PBMC proliferation (Figures 4(a) and 4(b)) and NK cell activation (FasL expression) in the presence of IL-2 or IL-15 (Figures 4(d), 4(f), and 4(h)) but maintained the NK population (Figures 4(c), 4(e), and 4(g)) in PBMC culture. In contrast, anti-*γ*c antibody could suppress both IL-2 triggered cytotoxicity against tumor cells (Figures 5(a) and 5(b)) and soluble factor release (Figure 5(d)–5(i)) when compared to anti-CD25 treated groups. The inhibitory effects of IL-15-induced soluble factor secretion were comparable between anti-*γ*c and anti-CD122 antibodies (Figure 5(j)–5(o) and *Supplementary 4*), while the antitumor effect was preserved (Figure 5(c)). These results clearly indicate the potential uses of anti-*γ*c antibody to treat NK cell-mediated autoimmune diseases.

### 3.7. *γ*c Cytokines Could Directly Activate Primary NK Cells Independent of Other Immune Cell Types

**Figure 6:**
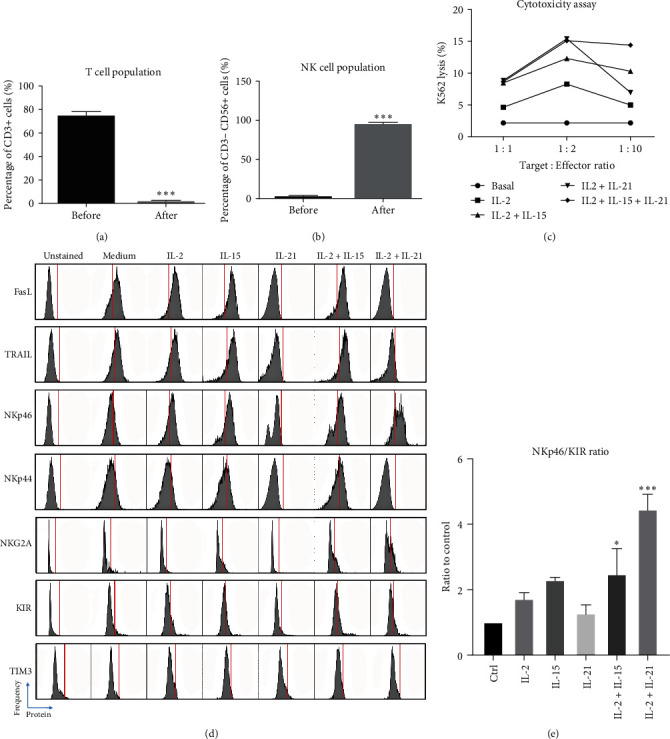
Long-term *γ*c cytokine treatment shaped autoimmune phenotype in purified NK cells. (a and b) Percentage of T cells and NK cells after expansion with IL-2 and IL-21.  ^*∗∗∗*^*p*  < 0.001 as compared to controls using student *t*-test, *N* = 2 for all groups. (c) Purified NK cell cytotoxicity assay to K562 cell line after priming and expansion with differential *γ*c cytokines for 14 days. (d) Representative histogram of FACS analysis showing differential levels of activating and inhibitory receptors after 3-day *γ*c cytokine (IL-2 : 50, IL-15 : 50, IL-21 : 25 ng/ml) treatment. (e) IL-2/IL-15 and IL-2/IL-21 combinatorial treatment significantly upregulated activating to inhibitory receptor ratio (NKp46/KIR).  ^*∗*^*p* < 0.05 and  ^*∗∗∗*^*p*  < 0.001 as compared to controls using Dunnett's multiple comparisons test following one-way ANOVA, *N* = 3 for all groups.

To conclude whether *γ*c cytokine could directly induce NK cell activation independent of other immune cell types, primary NK cells were purified, primed with IL-2, and expanded with the combined treatment of IL-2 and IL-21. After 14-day culture, T cells were completely depleted and NK cells were enriched to 90% in the culture (Figures 6(a) and 6(b)), with maintained cytotoxicity against K562 tumor following 1 day cytokine treatment (Figure 6(c)). NK cells were starved overnight and then incubated with a combination of *γ*c cytokines for 3 days. Results indicated that IL-2 or IL-15 but not IL-21 could induce expression of activating receptors, including FasL, TRAIL, and NKp46, on primary NK cell surface (Figure 6(d)). Interestingly, either IL-2/IL-15 or IL-2/IL-21 cotreatment groups showed higher NKp46 expression when compared to single cytokine-treated groups. The expression of inhibitory NKG2A and KIR was gently induced in all treatment groups except IL-2/IL-21 group. No expression of NK exhaustion marker TIM-3 was detected in any groups. To study the autoimmune phenotype of stimulated NK cells, activating/inhibitory receptor ratio was calculated by NKp46/KIR levels. The ratio clearly showed that primary NK cells were shifted to autoimmune phenotype in the presence of either IL-2/IL-15 or IL-2/IL-21 in the culture (Figure 6(e)).

### 3.8. Suppression of *γ*c Receptor Pathway-Attenuated NK Autoimmunity

**Figure 7:**
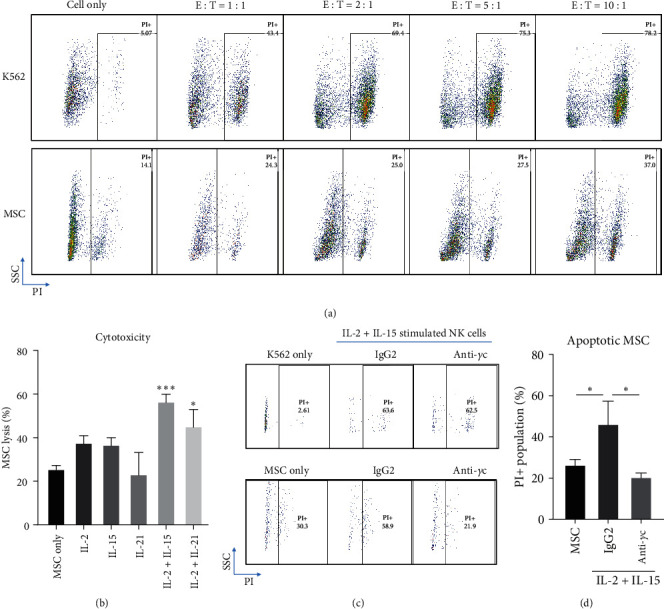
Anti-*γ*c reversed cytokines-induced autoimmune phenotype in purified NK cells. Purified NK cells were long-term stimulated with IL-2 + IL-21 (IL-2 : 50, IL-21 : 25 ng/ml) for 3 weeks, followed by cytotoxicity assay. (a) Representative images of FACS analysis demonstrating long-term stimulated NK cells with *γ*c cytokines exhibited cytotoxicity against K562 and MSC. Optimal cytotoxicity to MSC was obtained at E : T ratio 10 : 1. (b) Quantification demonstrated 3-day IL-2/IL-15 and IL-2/IL-21 (IL-2 : 50, IL-15 : 50, IL-21 : 25 ng/ml) combinatorial treatment promoted MSC lysis. (c) Representative FACS images showing anti-*γ*c reversed IL2-/IL-15-induced cytotoxicity to MSCs. (d) Quantification confirmed this result.  ^*∗*^*p*  < 0.05 and  ^*∗∗∗*^*p*  < 0.01 were compared to control using Dunnett's multiple comparisons test following one-way ANOVA. *N* = 3 for all groups.

As blockade of *γ*c cytokine pathway in T cells is proposed to be one of the ways to reverse development of autoimmunity [8, 71], the similar phenomenon was also studied in purified NK cells. Cultured NK cells acquired exaggerated cytotoxicity against K562 tumor cells, with development of autoimmunity against healthy MSCs simultaneously in the presence of IL-2 and IL-21 (Figure 7(a)). To further demonstrate this phenomenon, NK cells were starved overnight and challenged with different combination of *γ*c cytokines for 3 days. Results indicated that IL-2/IL-15 and IL-2/IL-21 pretreated groups showed significant autoimmunity against MSCs, which are consistent to the expression of activating receptors (Figure 7(b)) and intracellular levels of cytotoxic soluble factors (*Supplementary 4*) in the NK cell culture. More importantly, the presence of anti-*γ*c antibody could rescue MSCs during coculture with IL-2/IL-15 prestimulated NK cells (Figures 7(c) and 7(d)), suggesting that suppression of *γ*c activity on NK cells might be a potential way to treat autoimmune diseases.

## 4. Discussion

The present data support the phenomenon that *γ*c receptor signaling can modulate NK cell functions and phenotypes. Although IL-2 and IL-15 bind to individual receptor subunit, respectively, they use the common *γ*c and IL-2R*β* chains to trigger tyrosine phosphorylation of STAT3 and STAT5 via JAK1/JAK3 pathways [72]. Despite the similarities in the signaling cascades after receptor trimerization, IL-2 and IL-15 are responsible to trigger distinctive functions on several immune cells due to the differences in IL-2R*α* and IL15R*α* composition on self- and neighboring cell surfaces [73]. IL-2 is responsible to expand CD4+ helper T cells and regulatory T cells, while IL-15 could support the development of NK cells and central memory T cells [74]. For instance, IL-2 and lL15 can induce and both activate NK cell but IL-15 is more potent to trigger its survival and cytolytic activity [75]. Furthermore, IL-2 activated NK cells that undergo apoptosis upon initial interaction with endothelial and tumor cells, while IL-15 maintains NK cell survival under the same condition [76]. Although short-term IL-15 treatment could support NK cell function and survival in the culture, continuous IL-15 treatment eventually leads to decreased viability, functional impairment, and NK cell exhaustion in PBMC culture and *in vivo* xenogeneic model [77]. As a more recently discovered *γ*c cytokine, IL-21 is found to modulate NK progenitor cell development and proliferation [78]. Also, IL-21 could directly trigger NK cell-mediated cytotoxicity against breast cancer cells [79]. Therefore, it is important to dissect the overlapping and distinct relationship of a combination of *γ*c cytokines in NK cell homeostasis and autoimmunity. Currently, our *in vitro* data are consistent to previous findings that IL-2 and IL-15 specifically induce NK activation and cytotoxicity against target cells, while IL-21 preferably stimulates NK proliferation [80–82].

Among all studied cytokines, IL-15 acts as a more potent mediator to trigger NK cell activation and cytotoxicity as previously shown [75, 83]. These results might be explained by different expression of surface receptors on NK cells. IL-2 is responsible to upregulate the expression of activating NKG2D and DNAM-1, which recognize the stress-induced ligands on target cells [84]. IL-15 is more effective in upregulating the expression of NKp46 and NKp30, which are involved in the recognition and killing of tumor- and virus-infected cells; IL-15 also increases the CD69 expression, which is an early activation marker on T and NK cells [85, 86]. More importantly, synergistic effects in NK activation were shown in IL-2/IL-15 and IL-15/IL-21 cotreatments as previously reported [80–82, 87–89], demonstrating the high innate immune efficiency of NK cells responding to multiple stimulus.

NK cells can be characterized into NK1 and NK2 subpopulation according to cell surface markers and cytokine secretion profile as shown in T-helper 1 and 2 phenotypes in T cells. NK1 cell is classified by CD56^dim^/CD95^high^/perforin^high^ phenotype [90, 91] and actively produces IFN-*γ* and TNF-*α*; NK2 cell refers to CD56^bright^/CD122^high^/CD27^high^/CD69^high^ population [91] and secrets IL-5 and IL-13 [61, 92]; NK3 cell might express high level of CD127 and secretes TGF-*β* [61, 91] and NKr1 cell is reported to secrete IL-10 to promote immune regulation [61]. In the current study, IL-2 and IL-15 stimulation was identified to preferentially shift NK cell from NK0 (inactivated status) to a dominant NK1 phenotype (Figures 1 and 2 and *Supplementary 1*), which could potentially lead to long-term inflammation and development of autoimmunity in the culture [93]. On the other hand, IL-15/IL-21 cotreatment led to a mixed NK1/NKr1 phenotype, which might potentially explain IL-21 to exhibit immunosuppressive action on NK cell activation [94].

As aforementioned, *γ*c cytokines share common JAK/STAT pathways for signal transduction and immune responses in most immune cells. It is hypothesized that suppression of JAK/STAT pathway through blocking JAK protein or upstream *γ*c receptor subunits can potentially attenuate *γ*c cytokine-driven NK cell homeostasis as a strategy to tackle NK cell-mediated autoimmune or inflammatory diseases. Administration of JAK1/3 inhibitor tofacitinib, and antibodies targeting receptor subunit or *γ*c receptor, could suppress NK cell activities after IL-2 and IL-15 challenges (Figures 4 and 5). However, the normal immune functions against tumor cells by NK cells and cytotoxic T cells were impaired after tofacitinib treatment and so increased risk of cancer is usually observed after JAK inhibitor treatment [95, 96]. Alternatively, neutralization of CD25, CD122, or *γ*c receptor by respective antibodies could still block *γ*c cytokine-induced NK cell activity, while maintained IL-15 induced antitumor activity (*Supplementary 1* and *2* and Figures 4 and 5). A possible explanation for this observation is in accordance with other groups that complete loss of JAK1 activity in mouse NK cells lead to innate immune deficiency and reduced number of NK cells [97, 98]. Also, loss of JAK1 abrogates all downstream signaling pathways, while loss of JAK3 only suppresses STAT5 phosphorylation only [99, 100]. Taken together, we summarize that blockade of JAK3 activation through anti-*γ*c antibody treatment is enough to suppress *γ*c cytokine-driven NK cell activation while preserve the normal innate functions such as antitumor activity.

Although the blockade of JAK3 activity can inhibit NK cell activation, it is still unclear if *γ*c cytokine-driven NK1 phenotype plays a protective or detrimental role during the progression of autoimmunity. To answer this question, NK cells were purified from PBMC culture, expanded, and cocultured with MSCs to study NK cell-mediated autoimmunity as previously described (73). The current data suggest that IL-2/IL-15 and IL-2/IL-21 costimulation groups induced NK cell autoimmune phenotype (Figure 6) and cytotoxicity against healthy MSCs (Figure 7(b)), while pretreatment with anti-*γ*c antibody in NK cell cultures could rescue the MSCs (Figure 7(c)). These data suggest that *γ*c cytokine-driven NK1 phenotype might play a detrimental role in autoimmune diseases and the corresponding autoimmunity could potentially be reversed through blockade of JAK3 activity. However, the detailed relationship between NK cell activation and development of autoimmunity remains to be studied *in vivo*.

In humans, upregulation of *γ*c cytokines is usually observed together in various autoimmune diseases, including multiple sclerosis, celiac disease, and vitiligo. The current study suggests that IL-15 might be the major *γ*c cytokine to induce NK cell activation in those patients. For example, IL-15 is overexpressed in the intestinal areas of patients with inflammatory bowel disease (IBD) [101]. It is demonstrated that intestinal secretion of IL-15 could trigger NK cell-mediated small intestinal inflammation [102]. Prolonged IL-15 treatment to isolated human intestinal intraepithelial lymphocytes leads to massive production of IFN-*γ* and IL-10, eventually resulted in enhanced cytotoxicity against tumor cells [103]. Transgenic mice with intestinal IL-15 overexpression show distinct increase in the numbers of butyrate-producing bacteria, resulting in high susceptibility to dextran sulfate sodium (DSS)-induced colitis [104]. Consistently, IL-15 knockout mice were resistance to DSS-induced colitis, reduced NK cell population and IFN-*γ* level in lamina propria [105]. More importantly, IL-2 and IL-21 with their receptor subunits are also upregulated in patients diagnosed with IBD [46, 106–109], demonstrating that blockade of multiple *γ*c cytokines through specific inhibition of JAK3 signaling cascade via anti-*γ*c antibody might serve as a potent and safer strategy to treat autoimmune diseases with *γ*c cytokine upregulation. Future studies are required to test whether inhibition of NK cell activity is beneficial in certain autoimmune diseases, such as IBD, *in vivo*.

## 5. Conclusion

The current study provides a comprehensive comparison and characterization of *γ*c cytokines in NK cell functions, in which IL-2 and IL-15 stimulate the activated NK1 phenotype while IL-21 preferentially triggers NK cell proliferation. The results are further confirmed with purified NK cells. Specific inhibition of JAK3 signaling cascade by anti-*γ*c antibody could eliminate hyperactivated NK cells and suppress NK autoreactivity in the culture, which may serve as a potent and safer strategy to treat autoimmune diseases.

## Figures and Tables

**Table 1:** Reagent and antibody lists.

Category	Item	Company	Catalog number
Cytokine	Human IL-2	Sino Biological	11848-HNAH1-E
	Human IL-4	Sino Biological	11846-HNAE
	Human IL-7	Sino Biological	11821-HNAE
	Human IL-9	Sino Biological	11844-H08B
	Human IL-15	Sino Biological	10360-HNCE
	Human IL-21	Sino Biological	10584-HNAE
	Human premium grade IL-2	Miltenyi Biotec	130-097-748

Inhibitor	Tofacitinib (JAK1/JAK3)	Tocris (Abingdon, UK)	4556
	Ruxolitinib (JAK1/JAK2)	Tocris	7064
	Ritlecitinib (JAK3)	Tocris	6506

Neutralizing antibodies	Antihuman *γ*c antibody	R&D System, Minneapolis, USA	MAB2842
	Antihuman CD25 antibody	R&D System	MAB223
	Antihuman CD122 antibody	R&D System	MAB224
	Mouse IgG2b control	Thermo Fisher Scientific	02-6300

Flow cytometry antibodies	Antihuman CD3 antibody	BioLegend	317314
	Antihuman CD56 antibody	BioLegend	392414
	Antihuman NKG2A antibody	BioLegend	375104
	Antihuman NKG2D antibody	BioLegend	320808
	Antihuman FasL antibody	BioLegend	306407
	Antihuman KIR antibody	BioLegend	339504
	Antihuman NKp44 antibody	BioLegend	325110
	Antihuman NKp46 antibody	BioLegend	331922
	Antihuman granzyme B antibody	BioLegend	372206
	Antihuman perforin antibody	BioLegend	353312
	Antihuman IFN-*γ* antibody	BioLegend	502512

WB antibodies	Anti-STAT3	CST	9139
	Anti-p-STAT3	CST	9145
	Anti-STAT5	CST	94205
	Anti-p-STAT5	CST	9359
	Anti-STAT6	CST	5397
	Anti-p-STAT6	CST	56554
	Antitubulin	Sino Biological	100109-MM05T
	HRP goat antimouse IgG	CST	7076
	HRP goat antirabbit IgG	CST	7074

## Data Availability

The raw data supporting the statements in this article will be provided by the authors upon request.

## Supplementary Materials

Supplementary 1 IL-2 and IL-15 promoted Th1/NK1 phenotype in PBMC culture. Supernatant of *γ*c cytokines-treated PBMC culture was harvested for ELISA experiments.

Supplementary 2 Anti-CD25 and anti-CD122 suppressed IL-2- and IL-15-induced proliferation and activation.

Supplementary 3 Anti-CD25- and anti-CD122-suppressed IL-2- and IL-15-induced secretion of cytotoxic factors in PBMC culture.

Supplementary 4
*γ*c cytokines induced NK cell function and activation.
