# Supplementary figures and images for: Suppression of NK Cell Activation by JAK3 Inhibition: Implication in the Treatment of Autoimmune Diseases
Source: J Immunol Res. 2023 Dec 8;2023:8924603. doi: 10.1155/2023/8924603 (PMC10723930; doi:10.1155/2023/8924603)

A

IL-4

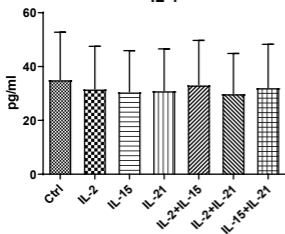

B

IL-5

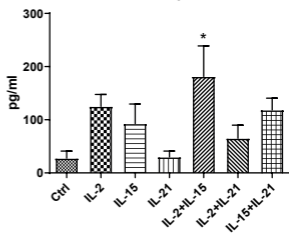

C

IL-13

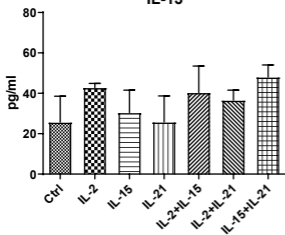

D

IL-10

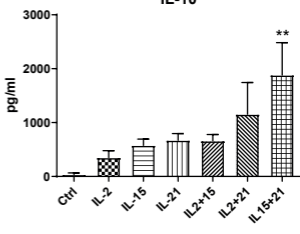

E

IFN- $\gamma$ /IL-5 ratio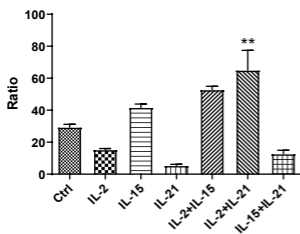

F

IL-6/IL-5 ratio

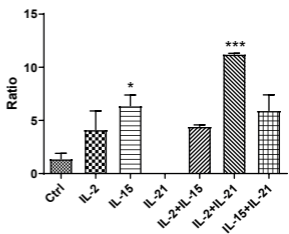

Supplement: Supplementary 1 — IL-2 and IL-15 promoted Th1/NK1 phenotype in PBMC culture. Supernatant of γc cytokines-treated PBMC culture was harvested for ELISA experiments. [file 8924603.f1.pdf]

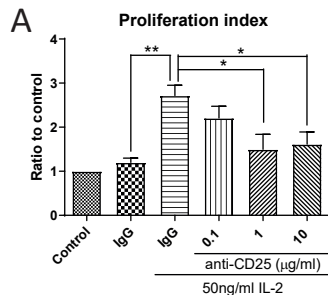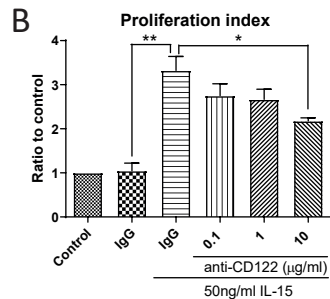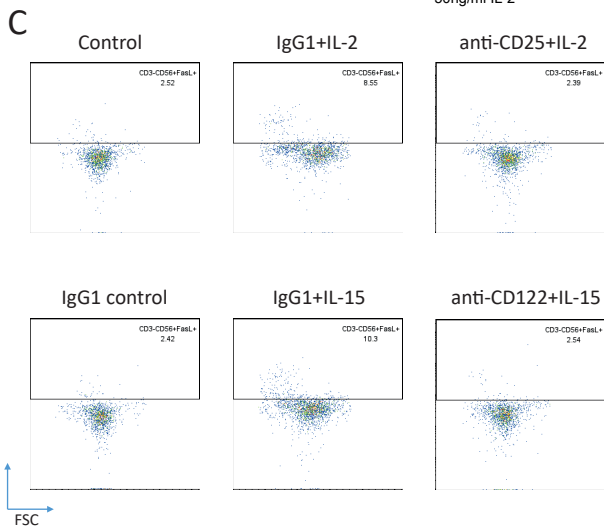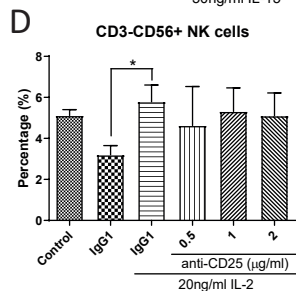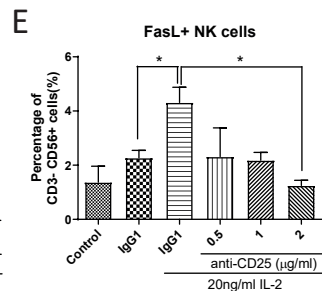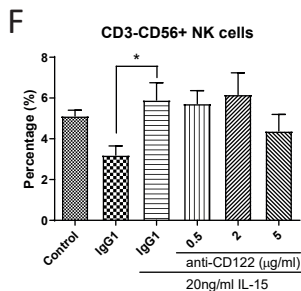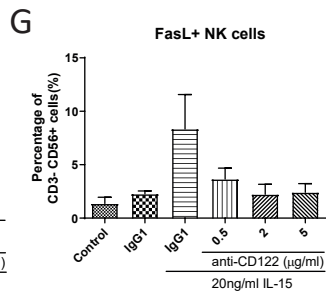

Supplement: Supplementary 2 — Anti-CD25 and anti-CD122 suppressed IL-2- and IL-15-induced proliferation and activation. [file 8924603.f2.pdf]

A

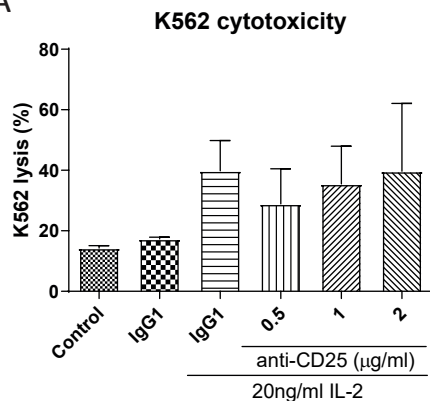

B

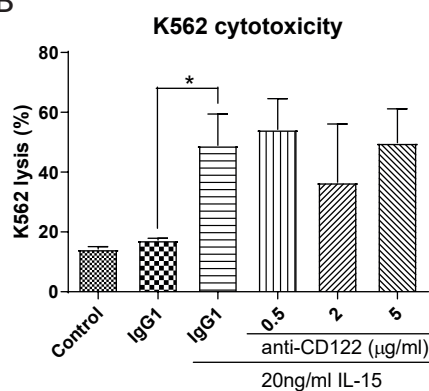

C

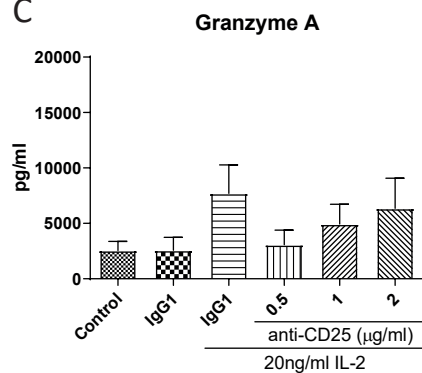

D

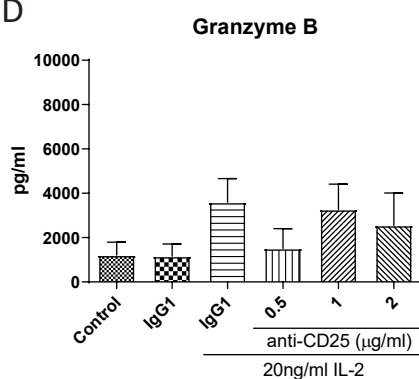

E

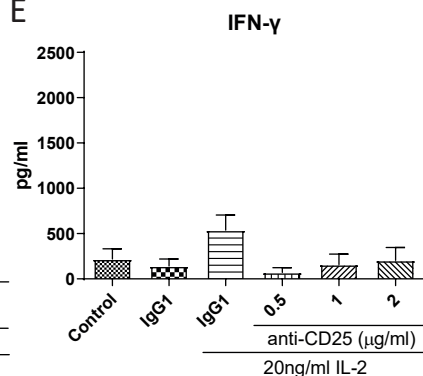

F

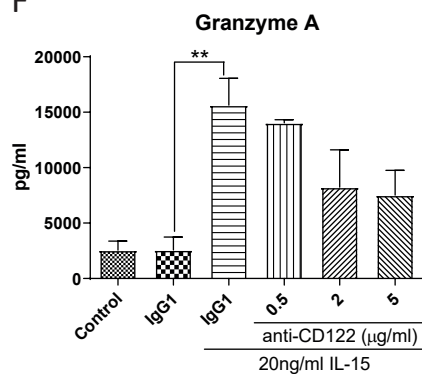

G

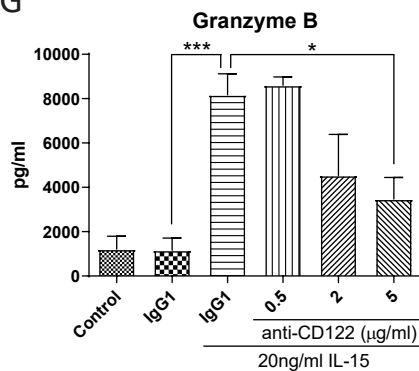

H

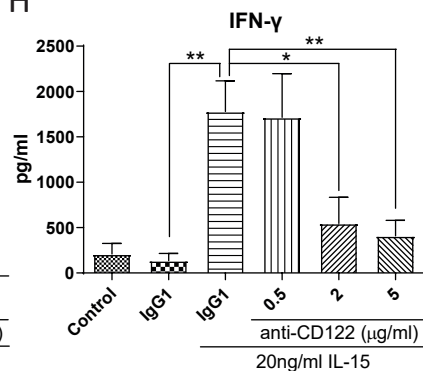

Supplement: Supplementary 3 — Anti-CD25- and anti-CD122-suppressed IL-2- and IL-15-induced secretion of cytotoxic factors in PBMC culture. [file 8924603.f3.pdf]

A

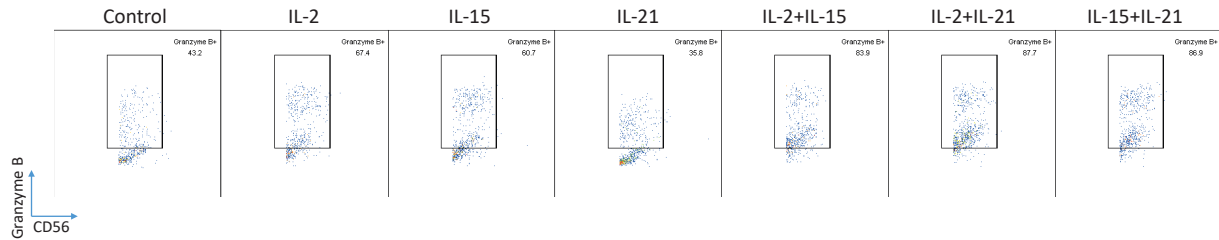

B

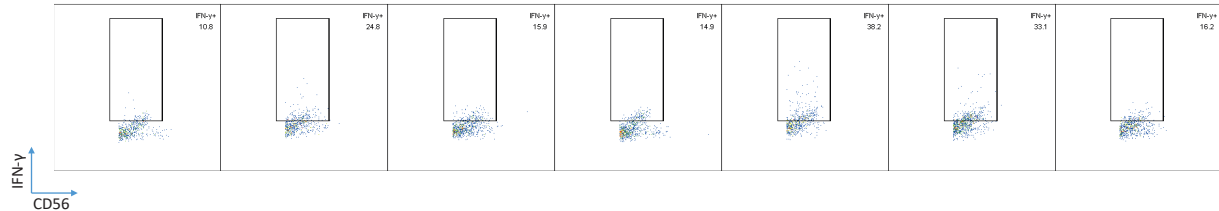

C

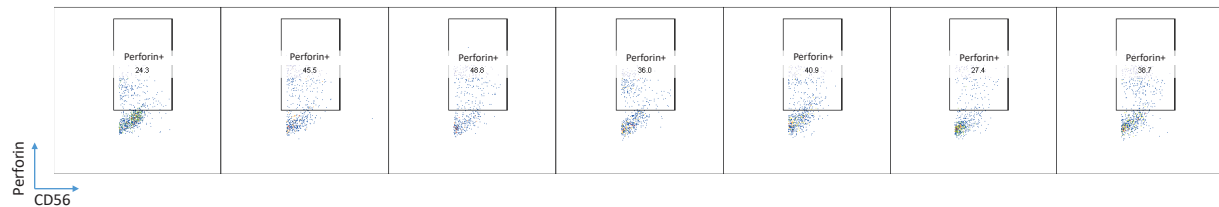

Supplement: Supplementary 4 — γc cytokines induced NK cell function and activation. [file 8924603.f4.pdf]
